# Correlation of protein binding pocket properties with hits’ chemistries used in generation of ultra-large virtual libraries

**DOI:** 10.1007/s10822-024-00562-4

**Published:** 2024-05-16

**Authors:** Robert X. Song, Marc C. Nicklaus, Nadya I. Tarasova

**Affiliations:** 1 Cancer Innovation Laboratory, Center for Cancer Research, National Cancer Institute, National Institutes of Health, Frederick, MD 21702 USA; 2 Computer-Aided Drug Design Group, Chemical Biology Laboratory, Center for Cancer Research, National Cancer Institute, NIH, Frederick, MD 21702 USA

**Keywords:** Drug discovery, Chemical reactions, Virtual screening, Protein pockets, Druggability, Transforms

## Abstract

Although the size of virtual libraries of synthesizable compounds is growing rapidly, we are still enumerating only tiny fractions of the drug-like chemical universe. Our capability to mine these newly generated libraries also lags their growth. That is why fragment-based approaches that utilize on-demand virtual combinatorial libraries are gaining popularity in drug discovery. These *à la carte* libraries utilize synthetic blocks found to be effective binders in parts of target protein pockets and a variety of reliable chemistries to connect them. There is, however, no data on the potential impact of the chemistries used for making on-demand libraries on the hit rates during virtual screening. There are also no rules to guide in the selection of these synthetic methods for production of custom libraries. We have used the SAVI (Synthetically Accessible Virtual Inventory) library, constructed using 53 reliable reaction types (transforms), to evaluate the impact of these chemistries on docking hit rates for 40 well-characterized protein pockets. The data shows that the virtual hit rates differ significantly for different chemistries with cross coupling reactions such as Sonogashira, Suzuki–Miyaura, Hiyama and Liebeskind–Srogl coupling producing the highest hit rates. Virtual hit rates appear to depend not only on the property of the formed chemical bond but also on the diversity of available building blocks and the scope of the reaction. The data identifies reactions that deserve wider use through increasing the number of corresponding building blocks and suggests the reactions that are more effective for pockets with certain physical and hydrogen bond-forming properties.

**Supplementary Information:**

The online version contains supplementary material available at 10.1007/s10822-024-00562-4.

## Introduction

Screening of virtual libraries of synthesizable compounds has become an increasingly important step in drug discovery [1, 2]. The surge in utilization of computational approaches has been stimulated by improvements in binding energy calculations, the growth of computational resources, advances in protein structures determination and availability of large and diverse virtual libraries of compounds [3–15]. However, our ability to access the vast druggable chemical space is still limited and will be impacted by the limits of computing resources for the foreseeable future [16–19]. We have the potential of generating trillions or more of virtual synthesizable molecules, but enumerating these chemical spaces, and thus converting them into screenable files is impractical if not impossible in practice. That is why fragment-based approaches that enumerate only parts of the chemical spaces, thus generating on-demand virtual combinatorial libraries are widely used [10, 20–22]. Most fragment-based methods identify synthetic blocks binding to sub-pocket(s) of a larger protein pocket first and then virtually “synthesize” libraries containing these blocks [8, 10, 23]. However, many different chemistries can be used for combining a particular set of blocks, and there are currently no time- and resources-saving guidelines for selection of reactions. To evaluate the impact of chemistries on the number of hits obtained through virtual docking, we have used the recently generated Synthetically Accessible Virtual Inventory (SAVI). SAVI comprises nearly 1.75 billion virtual molecules, each with a proposed synthesis scheme. It was constructed from 155,129 building blocks provided by Enamine (Kyiv, Ukraine, enamine.net) using robust chemistries encoded in 53 transforms [24]. SAVI transforms were written into rules based on an adaptation and extension of the CHMTRN/PATRAN programming languages describing chemical synthesis expert knowledge [25], which were originally developed in the context of the LHASA project [26]. We point out that the version of SAVI used for this study, SAVI-2020, consists of only single-step reactions, i.e. follows the scheme R1 + R2-> P (R1, R2: reactants; P: product). We note the terminology used here: We call the general reaction type (typically a "named reaction") a "chemistry" in the context of SAVI, whereas the individual CHMTRN/PATRAN rules are called "transforms." For example, SAVI uses the Suzuki–Miyaura cross-coupling chemistry, which is expressed in 6 different transforms (bromo, iodo, alkene cross-coupling etc.). Transforms have a descriptive name but also a four-digit number, which will frequently be used in the following. All 53 transforms can be downloaded from [27]. The cheminformatics toolkit CACTVS [28] was used to apply these rules for the virtual synthesis of the entries. By now, 169 SAVI compounds have been synthesized by us, Enamine LLC and the Medicinal Chemistry group of the National Center for Advancing Translational Sciences, NIH. These are the SAVI molecules whose syntheses we are aware of. Since the entire SAVI database can be freely downloaded without user registration, more SAVI compounds may have been synthesized elsewhere without our knowledge. SAVI’s predictions of synthetic accessibility were found to be more than 95% accurate. Enamine provides a database called REAL, which could be called a "sister" of SAVI since it is constructed from essentially the same set of building blocks. The mutual overlap between these two ultra-large databases, which in 2020 had somewhat over 1 billion molecules each, was only about 10%. Due to the essentially identical building block sets used for SAVI vs. REAL, the only plausible explanation was the difference in chemistries applied for the generation of the entries [24]. Unexpectedly, we found significant differences in the number of virtual hits when docking approximately equal-sized SAVI or REAL diversity sets (each about 3 million compounds in size) into the same protein pocket. This observation provided the impetus for this study. Since these two databases were constructed from the same building blocks, and the major differences were in the reactions used, we hypothesized that linking chemistries may favor certain pockets but not the others.

A systematic investigation of the impact of chemistries on the number of virtual hits is particularly important as the ultra-large libraries continue to grow rapidly. We have expanded the number of reliable transforms that can be used for SAVI generation to more than 120. The number of commercially available synthesis building blocks has also increased. Enamine alone has now 1 billion made-on-demand blocks (MADE) [29]. Consequently, the next version of SAVI could represent trillions of molecules. The expansion of the accessible chemical space is a welcome trend that is likely to improve and accelerate drug discovery. However, enumeration and screening of such databases and their use in their entirety will remain impossible for the foreseeable future. Enumeration of only specific parts of accessible chemical space (so-to-speak the "optimal chunks" of the space) that maximize success in screening is therefore likely to continue to be widely used in the future. Consequently, to enumerate the most appropriate part of the chemical space for a given target, it would be helpful to know not only which building blocks are better suited for the target pocket, but also which coupling chemistries are more likely to generate high-scoring virtual hits.

To evaluate the possible correlation between pocket properties and transforms used for library generation, we performed docking of a SAVI diversity set, containing about 3 million structures collected from all 53 transforms listed in Table 2, into 40 protein pockets (Table 1). We conducted the analysis around the chemistries generating the compounds rather than structural motifs they produce because of practical considerations: During the generation of SAVI, filtering by the CHMTRN reaction logic occurs, which for SAVI-2020 excluded about 50% of the initially formed reactant pairs because the proposed structures could not be made in a one-step reaction. This filtering influences the set of resulting structures and their ability to interact with proteins. Thus, the sets of compounds containing the same structural motif generated by different synthetic methods will be different because of different exclusion rules in the individual transforms. This is why we analyze the chemistries separately, focusing on the reaction rather than on the structural motif it produces.

**Table 1:** Pocket types: small molecule druggable pockets (SM) and protein–protein interaction interfaces (PPI)

PDB	Target	Pocket Type	Volume, Å^3^	DLID	Number of virtual hits**	RMSD, Å***	Redocking score
1sj0	ESR1	SM	598	1.23	9818	0.49	− 56.4
3kl6	FA10	SM	424	0.16	5244	0.003	− 38
5dwr	PIM1	SM	764	0.63	1109	0.3	− 34.9
3ruk	CP17A	SM	683	1.73	774	0.00	− 32.0
6gt3	A2A	SM	771	1.56	14,387	0.40	− 34.3
3odu	CXCR4	SM	619.1	0.8	458	0.91	− 27.0
4mbs	CCR5	SM	523.1	0.15	111	0.36	− 32.6
3lpb	JAK2	SM	1064	1.22	1911	0.61	− 32.8
2owb	PLK1	SM	859	1.06	66,418	0.64	− 40.4
7khk	KIT	SM	469.3	0.55	11,147	0.68	− 45.5
5i96	IDHP	SM	451.9	1.45	2059	1.12	− 26
5ef8	HDAC6	SM	325	0.64	279	0.92	− 32
4tvj	PARP2	SM	792.8	0.57	7541	0.54	− 55.1
5fhz	ALDH1A3	SM	1060	1.41	4026	0.86	− 21
2oj9	IGF1R	SM	640.2	0.31	892	0.58	− 37.8
4xe0	PK3CD	SM	296.8	0.38	155	0.00	− 28
3d4q	BRAF	SM	741.4	0.73	13,715	0.00	− 33.2
5vv0	NOS1	SM	707.5	0.64	871	1.13	− 18.2
6tz7	calcineurin	SM	925.8	0.5	13,889	0.8	− 54.2
5kj2	p300	SM	599.9	0.76	608	0.19	− 30
4ivd	JAK1	SM	1210	0.99	6063	0.54	− 35.3
5gmh	TLR7	SM	596.8	0.8	3414	0.39	− 49.9
4ixd	ITGAL	SM	413	− 0.1	3753	0.67	− 29
1qw6	NOS1	SM	315	0.04	399	0.33	− 49.7
4ziaB	STAT3 ND	PPI	561	− 0.5	1295		46
6m0jA	SARS CoV2 Spike	PPI	474.7	0.38	349		
4lvt	BCL-2	PPI	572.6	0.27	1971	0.48	− 33.3
5lof	MCL1	PPI	307.9	0.76	40	0.83	− 38
5v52	TIGIT	PPI	108	− 0.7	326		
5wlb	K-Ras	PPI	339.9	0.18	11,213		
5wha	K-Ras	PPI	450	0.58	6102		
6dhb	TIM-3	PPI	297.1	− 0.5	163		
5v1y^22^	Rpn13	PPI	328	0.13	4284		
4lwv	MDM2	PPI	289	0.19	78	0.42	− 33.2
7rpz	KRAS	PPI	420	0.94	1003	0	− 45
4lxd	BCL-2	PPI	132.1	− 0.6	375	0.42	− 37.5
6h6q	XIAP	PPI	291.3	− 0.2	1	0.43	− 33.3
6o5i	MEN1	PPI	949	0.33	229	0.39	− 39
7p5e	KEAP1	PPI	1007	0.68	850	0.37	− 38.2
5n2f	PDL1	PPI	1323	1.02	2034	0.79	− 36.2

Addition of a capital letter to the PDB ID (such as "A", "B") denotes the protein subunit/chain used ^*^DLID: Drug-like density of the pocket

^**^Number of hits obtained by virtual screening of 2,955,416 compounds of SAVI diversity set

^***^Ligands present in the structures of the complexes were docked into the corresponding protein pocket and the docking pose was compared to the experimental structure

## Results and discussion

Target pockets (Table 2) were selected from PDB to represent two pocket types: small molecule (SM) pockets and protein–protein interaction (PPI) pockets. The majority of selected pockets bind well-characterized ligands that have advanced into the clinics. However, we also included several less studied but interesting and potentially impactful pockets that either are difficult to target, or which represent surfaces involved in protein–protein interactions. PPI-based inhibitors and modulators are the type of therapeutics that the scientific community is increasingly aiming at. To ensure that the structures were suitable for virtual screening, the ligands present in the chosen complexes were redocked into the corresponding pockets, and only structures that allowed for correct prediction of ligand poses were included in the analysis. For docking and virtual screens, we used the ICM-Pro software (Molsoft, San Diego, CA). Although the software has been benchmarked before [30–32], we have evaluated the correctness of docking poses for the pockets with known ligands (Table 1). Most of docked complexes had RMSD < 1 Å when compared to the experimental structures. In two cases where RMSD exceeded 1 Å (PDB:5i96 and 5vv0), all the differences were in the part of the molecules exposed to the solvent, while poses inside the pocket were determined with high accuracy. Although the structures used appeared to predict binding correctly when tested with “native” ligands, the presence of false positives in virtual screens is inevitable [33]. Testing binding properties experimentally for all identified virtual hits was not possible in the context of this study. We were able to verify binding for several top virtual hits for eight targets, which are currently studied in our lab, two of which have recently been published [34, 35]. It is reasonable to suggest, however, and the data generated for the eight targets confirms, that percentage of noise or false positives for a particular pocket is distributed evenly across the database and does not depend on the transform. Consequently, virtual hit rates can serve as surrogates of number of binders.

**Table 2:** Docking hits rates for SAVI-2020 transforms applied in the generation of the diversity set used for docking into 40 protein pockets

ID	Name	Scheme	Virtual hits rate, %	Number in the set used for docking	Number in SAVI
1031	Paal-Knorr Pyrroles synthesis		0.47	32,785	65,570
**1039**	Feist Synthesis of Pyrroles		0.97	**1437**	1437
1171	Hantzsch Thiazole Synthesis		5.2	9423	94,336
**1391**	[2 + 2]-Cycloaddition of Allenes to Alkenes		0.000	**20**	20
**1439**	Pyrazoles from Beta Carbonyl Carboxylic Acid Derivatives		**0.07**	21,137	42,275
2201	Fused Arylpyridines via o-Aminocarbonyls		1.7	57,453	582,318
**2218**	Tetrazoles from Azide and Nitriles		0.53	**4376**	4376
2230	Phthalazin-1-ones from 2-Acylbenzoic Acids		6.2	22,918	45,836
**2238**	Fused Aryl(2,3-H/R)Pyridines (Pictet-Spengler)		**0.04**	90,655	1,827,991
2267	Sonogashira Coupling		7.9	120,752	24,239,698
**2269**	Kabbe Synthesis of 4-Chromanones		**0.18**	14,642	146,610
2630	Benzazepin-2-ones by Pictet-Spengler Reaction		0.08	10,184	10,184
**2684**	Benzo[b]furans from 2-Hydroxyphenyl Acetylenes		0	**942**	942
2875	Copper[I]-catalyzed azide-alkyne cycloaddition		2.5	59,078	1,208,372
6003	Buchwald-Hartwig Ether Formation		10.0	86,370	43,731,278
6004	Suzuki–Miyaura Cross-Coupling (Bromo)		11.2	56,880	5,803,732
6005	Suzuki–Miyaura Cross-Coupling (Iodo)		9.5	39,814	804,723
6006	Suzuki–Miyaura Cross-Coupling (Chloro)		7.75	29,010	2,971,512
6008	Suzuki–Miyaura Cross-Coupling with Alkene		6.7	24,659	49,318
6009	Suzuki–Miyaura Cross-Coupling of Alkenes		4.18	43,535	876,832
**6013**	Hiyama Aryl-Alkenyl Cross-Coupling		1.08	**2966**	2966
6014	Hiyama Non-Aromatic Cross-Coupling		7.5	8976	8976
**6015**	Hiyama Allyl Cross-Coupling		0	**148**	148
6016	Hiyama Carbonylative Cross-Coupling		11.0	12,026	24,052
**6017**	Hiyama Cross-Coupling with Arylhydrazine		0.63	**1106**	1106
6022	Liebeskind-Srogl Thioamide Coupling		7.6	9113	91,767
**6024**	Liebeskind-Srogl Nitrile Formation		0	**541**	541
6025	Liebeskind-Srogl Heterocyclic Coupling		1.14	11,642	116,790
6026	Sulfonamide Schotten-Baumann		2.27	123,951	124,375,067
6027	Sulfonamide Schotten-Baumann from Sulfonate		2.9	66,826	6,803,351
6028	Sulfonamide Schotten-Baumann from Thiol		2.9	91,691	91,704,439
6029	Sulfonamide Schotten-Baumann from Aryl Bromide		3.2	105,957	211,944,731
6031	Mitsunobu Reaction		3.4	155,673	155,748,444
**6032**	Mitsunobu carbon–carbon bond formation		**0.02**	18,139	181,524
6033	Mitsunobu SN2' Reaction		7.8	8368	83,940
6034	Mitsunobu Imide Reaction		2.07	132,730	27,177,967
6035	Mitsunobu Aryl Ether Formation		2.9	84,542	42,306,237
6036	Mitsunobu Sulfonamide Reaction		2.97	104,307	10,589,664
6038	Ester or Amide or Thiolester Formation		3.95	183,070	366,293,581
6039	Williamson Ether Synthesis		2,67	103,046	103,177,836
6041	Buchwald-Hartwig Reaction—Amines		8.1	132,160	264,514,821
6043	Buchwald-Hartwig Reaction—Sulfonamides		8.87	160,097	32,762,479
7005	Benzimidazoles from o-Phenylenediamines		4.46	85,452	1,733,461
7009	Acylsulfonamide from Sulfonamide and Carboxylic Acid		6.42	92,318	46,207,962
7013	Benzimidazoles from o-Phenylenediamines and Aldehydes		3.92	77,938	1,575,305
7014	Benzimidazoles from o-Phenylenediamines and Aldehydes		6.92	43,989	888,165
7015	Sulfonamide from sulfonic acid and amine		3.17	47,678	4,856,868
7017	Sulfonamide alkylation with a cyclic ether		3.42	36,416	3,732,596
**7018**	Sulfonamide acylation		**0.146**	29,975	300,300
7019	Wittig Reaction		2.58	142,425	142,522,022
7020	Wittig via Methoxy-Ylide		1.39	11,557	11,557
7021	Horner-Wadsworth-Emmons Olefination		3.26	15,922	31,843
7022	Chan-Lam coupling		2.44	128,600	26,186,137

A virtual hit was defined as a compound with a docking score below −32 for pockets that had redocking scores for native ligands below −32

Score cutoffs equal to redocking scores of native ligands were used for the rest, as described in the Methods section

The transforms excluded from the analysis have their ID number bolded

If the reason for exclusion was due to low representation in the database (“starved” transforms), that transform will also have their numbers in the set value bolded and underlined

If the reason for exclusion was due to a low number of virtual hits across entire set of pockets, that transform will have their hit rate value bolded and underlined

We chose the SAVI diversity set containing 2,955,416 compounds for the exploration because of practical considerations. Docking the entire SAVI database into just one pocket would take more than 280 days when running 1000 parallel processes on the NIH supercomputer cluster. Docking of the diversity set into one pocket requires around 50,000 CPU Hours, which is doable on a computer cluster. Although docking of larger sets may allow for more sensitive detection of differences between different transforms, it would require prohibitively large computational resources when used for the multiple pockets that we aimed to evaluate for this study.

**Fig. 1:**
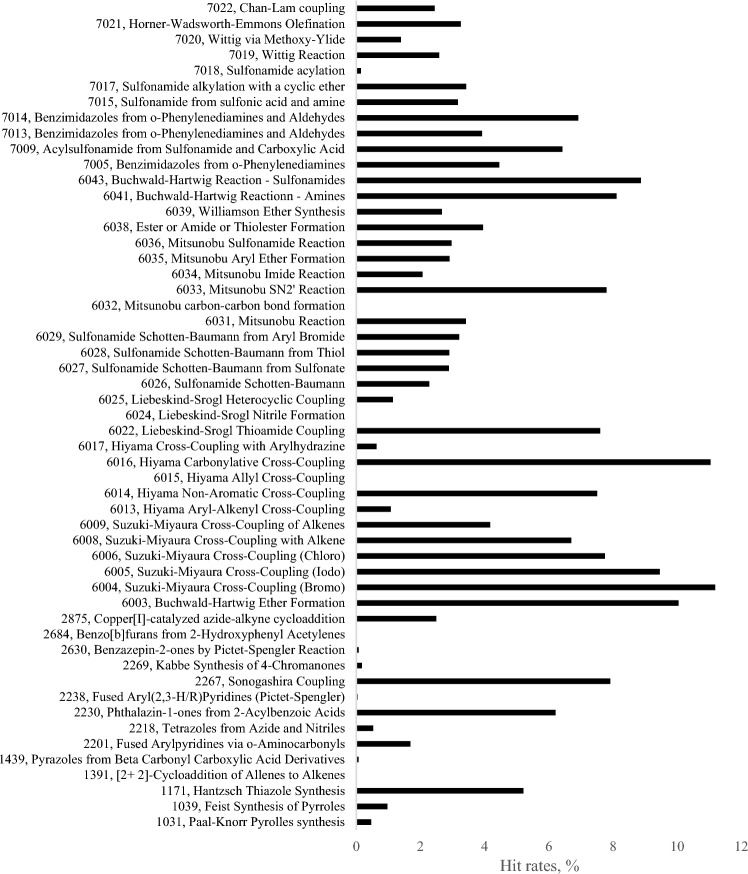
Virtual hit rates for 53 transforms used for SAVI generation. The hits were identified by docking 2,955,416 compounds of SAVI diversity set into 39 well characterized protein pockets. To compensate for differences in the occurrence rate of a particular transform in the diversity set, the total number of virtual hits for each transform has been normalized by dividing it by the number of compounds produced by the transform in the screening library

Remarkably, virtual hit rates across 40 targets differed significantly between different transforms (Fig. 1, Table 2). Several transforms had to be excluded from further analysis because they were represented by too few compounds in SAVI as well as in the diversity dataset. This underrepresentation occurs due to the low number of available synthetic blocks that are needed for these "starved" transforms. The following “starved” transforms were excluded from the analysis because they produced less than 10000 compounds for the entire SAVI: Feist synthesis of pyrroles (1039), [2 + 2]-cycloaddition of allenes to alkenes (1391), synthesis of tetrazoles from azide and nitriles (2218), benzo[b]furans synthesis from 2-hydroxyphenyl acetylenes (2684), Hiyama aryl-alkenyl cross-coupling (6013), Hiyama allyl cross-coupling (6015), Hiyama cross-coupling with arylhydrazine (6017) and Liebeskind-Srogl nitrile formation (6024) (Table 1). The number of available building blocks for each transform can be found at: https://www.nature.com/articles/s41597-020-00727-4/tables/7. Several transforms had sufficient representation in the database but could not be used for reliable evaluation because they produced too few virtual hits across all tested targets and zero hits for many of them. Transforms that had to be excluded for this reason were: pyrazole synthesis from beta carbonyl carboxylic acid derivatives (1439), synthesis of fused aryl(2,3-H/R) pyridines by Pictet-Spengler reaction (2238), Kabbe synthesis of 4-chromanones (2269), Mitsunobu carbon–carbon bond formation (6032), and sulfonamide acylation (7018). Thus, they may be less valuable for the current drug discovery efforts in general (Fig. 1, Table 1). The weak performance of some of these transforms can be attributed to the small number of compounds for one of the two building blocks needed by the transform. Although instances of the second type of blocks needed (R2) could be plentiful in the building block set and the number of generated compounds therefore relatively large, the overall diversity of the products is limited if the R1 subset consists of, say, fewer than hundred compounds (and those may be structurally closely related). Transforms 1439, 2269 and 6032 are examples of such cases: https://www.nature.com/articles/s41597-020-00727-4/tables/7. Remarkably, several chemistries produced subsets with very high virtual hit rates. Suzuki–Miyaura cross-couplings (6004, 6005 and 6006) were among the most productive ones. Interestingly, Suzuki–Miyaura coupling is among the most frequently used reactions in current medicinal chemistry [36]. Our data shows that this chemistry deserves the attention it receives. However, the most frequently used reaction, amide bond formation (transform 6038) [36], was less productive with a virtual hit rate that was roughly three times lower than that for Suzuki–Miyaura cross-couplings.

This should not be interpreted as a lower general usefulness of amides in drug discovery. This particular case emphasizes the differences in the impact of chemistries between traditional medicinal chemistry that employs multi-step synthesis and virtual libraries constructed using one- or two-step reactions. One of the possible reasons for the relatively poor performance of transform 6038 is suboptimal selectivity, which led to reduced scope, and exclusion of building blocks containing hydrogen bond donors that facilitate interactions with proteins, such as amino, carboxyl, hydroxyl and sulfonamide groups. In multi-step synthesis, protection/deprotection of these groups could preserve them, thus increase the protein interaction potential of the products. Selectivity of chemistries is likely to play a role in “productivity” of other transforms and it is an additional factor that needs to be considered during generation of custom libraries. Our data also suggest additional transforms that deserve efforts in expanding. For example, Hiyama carbonylative cross-coupling should be expanded by adding more aryl triethoxysilanes into the collection of the building blocks. Expanding the collection of arylboronic acids would benefit not only Suzuki–Miyaura cross-coupling, but also the highly productive Liebeskind–Srogl heterocyclic coupling (6025). It should be emphasized that the efficacy of a transform in producing potential virtual hits can depend not only on the properties/geometry of the bond it generates but also on reaction selectivity and diversity of the building blocks available. As discussed above, selectivity of the reaction allows to preserve the functional groups of the blocks that can be beneficial for protein binding while diversity increases the chances of finding a good fit for a particular pocket. Transforms 6004–6006 produce structurally similar di-aryl compounds through Suzuki–Miyaura cross-coupling. However, the virtual hit rates for 6004, which uses bromo aryl blocks, is about 40% higher than for 6005 or and 65% higher than for 6006 that use iodo and chloro aryls, respectively. The set of building blocks used for SAVI-2020 had a 7.3 times larger number of bromo aromatic compounds than iodo-derivatives (https://www.nature.com/articles/s41597-020-00727-4/tables/7), thus allowing for higher diversity in the products of transform 6004 vs. 6005. Chloro-aromatic blocks are even more numerous than the bromo-derivatives. However, the reaction is less selective for chloro compounds, which results in a 44 times higher number of excluded products, effectively reducing the number of useful blocks for transform 6006. The diversity of the blocks that can potentially impact virtual hit rates is likely to change with time along additional synthetic efforts in building blocks generation. Thus, virtual hit rates can be improved for less productive transforms in the future.

The pocket properties evaluated for potential impact on the number of virtual hits included volume, area, radius, hydrophobicity, nonsphericity, aromaticity, buriedness, drug-like density (DLID [37]), the numbers of hydrogen bonds donors, and the number of acceptors. The hydrogen bond forming potential of each pocket was evaluated manually. The rest of the parameters were determined using the PocketFinder function of ICM-Pro. The correlation of these properties with the number of virtual hits for each transform was determined for the entire SAVI diversity set.

**Fig. 2:**
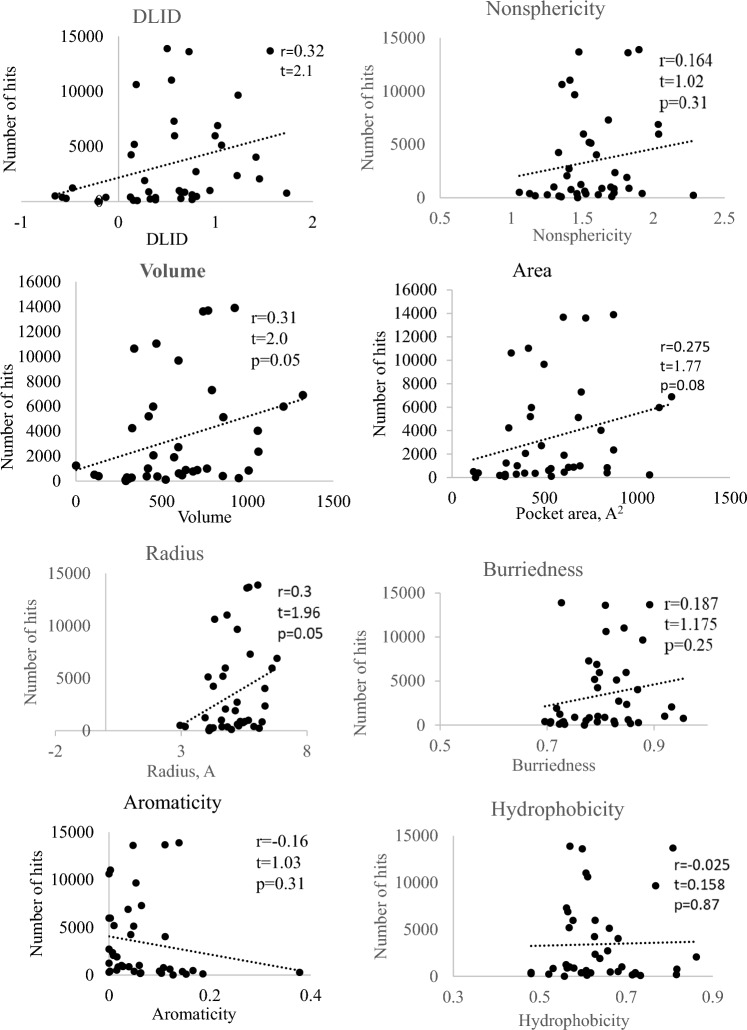
Total number of virtual hits generated by virtual docking of SAVI diversity set into protein pockets with different properties. The parameters for each property were determined using the PocketFinder function of ICM-Pro software (Molsoft). Dotted lines represent linear trends with corresponding correlation coefficient (r), Student's *t*-distribution, and p-values shown

The binding score produced by docking for every molecule is influenced by many factors. That is why we did not expect strong dependencies for any single parameter, but rather tendencies. That is why we include correlations that have p > 0.05. For the whole database, the number of virtual hits showed a statistically significant positive correlation (with p-value < 0.05) with properties related to pocket size: volume and radius (Fig. 2). Most of the pockets with high numbers of virtual hits had volumes between 300 and 1000 Å^3^, and virtual hit rates were significantly lower both below and above this range. Similarly, the graphs suggest that the most productive values of the radius are between 4 and 6.2 Å and between 300 and 900 Å^2^ for the pocket surface area. This can be explained by the size distribution of the database entries as it contains only limited numbers of molecules with MW < 200 and > 550 [24]. The degree of hydrophobicity of the pocket did not yield any definite trends. Surprisingly, aromaticity appeared to have negative correlation, although aromatic interactions have been suggested to contribute to ligand–protein binding [38, 39]. However, the correlation was not statistically significant.

**Fig. 3:**
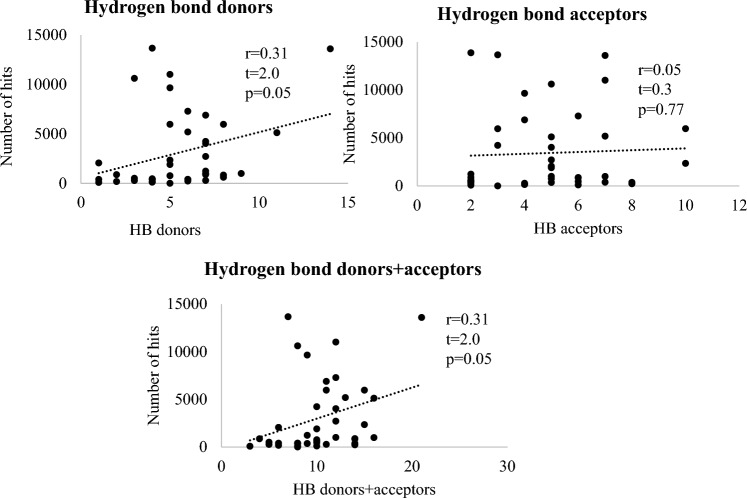
The impact of hydrogen bond-forming capacity of the pockets on the total number of virtual hits for the entire SAVI diversity set. The number of hydrogen bond donors had a positive, statistically significant, correlation with the number of docking hits

Nonsphericity and buriedness demonstrated positive correlation with the number of virtual hits (Fig. 2) but it was statistically insignificant for both parameters. The number of hydrogen bond acceptors (HBA) in the pocket did not show any significant correlation. In contrast, the number of hydrogen bond donors (HBD) had significant positive correlation with the number of docking hits (Fig. 3). The observed dependencies on HBD could be caused by prefiltering of the database building blocks for “drug-like” properties. Hydrogen bond acceptors of potential drugs are widely believed to be less detrimental than hydrogen-bond donors with regards to solubility, cell permeability and bioavailability [40]. Lipinski’s rule of 5 is more restrictive to hydrogen bond donors than to hydrogen bond acceptors allowing no more than 5 HBDs vs. up to 10 HBAs [41]. Consequently, the database will have more HBA-rich compounds that prefer HBD-rich pockets.

To compare the degrees of dependencies for different transforms, we used correlation coefficients (Tables 3 and S1). Correlations with pockets’ properties differ for different transforms (Table 3) and frequently have opposite signs. The relatively small number of pockets screened does not allow one to make statistically justified conclusions for many correlations as p-values fall short, sometimes just slightly above 0.05. The data shows that those differences do exist, and additional future screens will permit to establish comprehensive correlations. Nevertheless, several dependencies could be established. Pocket sizes showed positive correlations with virtual hit rates for all transforms, with transforms 1171, 2201, 6003, 6004, 6005, 7013, 7014 and 7022 showing the strongest correlations, suggesting that they could work better for larger pockets, but not for the small ones. Although the number of virtual hits increased with an increase of pocket buriedness and nonsphericity for the majority of transforms, only transform 2267 had statistically significant correlation with buriedness in this study.

**Table 3:** Pearson’s coefficients for correlations between protein pocket properties and the number of docking hits in SAVI diversity set

ID	Moiety formed	Volume	Radius	Area	DLID	Buriedness	Aromaticity	HB donors	HB acceptors
1031	Pyrroles	0.13	0.12	0.09	0.16	0.14	0.16	0.13	0.19
1171	Pyrroles	**0.36**	**0.36**	**0.33**	**0.39**	0.2	− 0.2	0.03	0.014
2201	Arylpyridines	**0.36**	**0.34**	**0.31**	**0.32**	0.23	− 0.27	0.14	0.25
2230	Phthalazin-1-ones	0.163	0.18	0.17	0.2	0.156	− 0.23	0.131	− 0.08
2267	Aryl-acetylenes	0.247	0.25	0.18	0.186	**0.32**	0.15	**0.31**	0.265
2875	Triazoles	0.1	0.04	0.07	0.196	0.22	− 0.28	**0.33**	**0.32**
6003	Aryl-ethers	**0.48**	**0.41**	**0.43**	**0.35**	0.13	− 0.11	0.22	− 0.05
6004	Di-aryls	**0.48**	**0.39**	**0.43**	**0.38**	0.2	− 0.22	0.23	0.03
6005	Di-aryls	**0.39**	**0.36**	**033**	**0.36**	0.2	− 0.2	0.24	0.06
6006	Di-aryls	**0.35**	0.25	**0.33**	0.27	0.17	− **0.31**	0.29	0.15
6008	Aryl alkenes	0.22	0.2	0.18	0.19	0.16	− 0.2	**0.36**	0.22
6009	Alkenes	0.08	0.09	0.04	0.19	0.22	− 0.2	0.05	− 0.05
6014	Dienes	0.12	0.16	0.1	0.19	0.15	0.15	**0.38**	0.25
6016	Diaryl ketones	0.17	0.14	0.15	0.2	0.14	− 0.11	**0.52**	0.17
6022	Aryl-dihydro-pyrrole	0.01	− 0.52	− 0.031	0.14	− 0.09	− 0.17	0.12	0.17
6026	Sulfonamides	0.24	0.17	0.17	**0.35**	0.27	− 0.14	**0.37**	0.012
6027	Sulfonamides	0.13	0.08	0.08	0.21	0.21	− 0.11	0.17	− 0.1
6028	Sulfonamides	0075	0.072	0.247	0.25	0.23	− 0.08	0.1	− 0.005
6029	Sulfonamides	0.185	0.13	0.12	**0.31**	0.27	− 0.163	**0.34**	0.05
6031	Esters	0.067	0.12	0.038	0.24	0.29	− 0.27	0.2	**0.33**
6034	Di-esters	0.029	0.09	0.01	0.19	021	− 0.2	0.18	0.15
6035	Aryl ethers	0.14	0.2	0.11	0.28	0.24	− 0.15	0.28	0.14
6036	Sulfonamides	0.02	0.108	0.01	0.22	0.21	− 0.11	0.1	0.03
6038	Esters, amides, thioesters	0.14	0.17	0.13	0.19	0.2	− 0.3	0.19	0.28
6039	Ethers	0.13	0.08	0.08	0.235	0.24	− 0.27	0.3	0.24
6041	Aryl amines	0.24	0.08	0.2	**0.35**	0.3	− 0.26	**0.39**	0.15
6043	Aryl sulfonamides	0.09	0.11	0.03	0.29	0.27	− 0.18	0.11	− 0.05
7005	Benzimidazoles	0.13	0.302	0.359	0.3`	0.3	− 0.3	0.06	0.12
7009	Acylsulfonamide	0.143	0.2	0.15	0.14	0.06	− 0.1	0.194	0.09
7013	Benzimidazoles	**0.31**	0.25	0.29	0.31	0.24	− 0.28	**0.35**	0.16
7014	Benzimidazoles	**0.31**	0.25	0.29	**0.41**	0.24	− 0.28	**0.353**	0.16
7015	Sulfonamides	0.24	0.25	0.21	0.28	− 0.03	− 0.09	0.279	− 0.08
7017	Sulfonamides	0.21	0.25	0.17	**0.33**	0.21	− 0.12	0.17	− 0.002
7019	Acyl sulfonamides	0.19	0.21	0.16	0.249	0.2	− 0.19	0.175	0.043
7020	Ketones	0.05	0.09	0.05	0.1	0.16	− 0.25	**0.33**	**0.35**
7021	Alkene asters	0.23	0.065	0.2	0.18	0.11	− 0.21	**0.55**	0.24
7022	Amines	**0.34**	**0.33**	**0.36**	0.15	0.0002	− 0.14	**0.17**	− 0.07

Statistically significant correlations with p-values below 0.05 are in bold and underlined

Table S1contains the full set of data with t- and p-values included

Aromaticity had negative, but insignificant, correlation for all transforms (Table 3). Although the number of hydrogen bond acceptors in the pocket did not show any definite correlation for the whole diversity set, it demonstrated strong positive correlation for transforms 2875 (copper[I]-catalyzed azide-alkyne cycloaddition), 6031 (Mitsunobu reaction) and Wittig ketone synthesis (7020). Transform 2875 produces heterocycles with hydrogen-donating properties that can explain this trend. For transform 6031 and 7020 the reason could be the properties of the blocks that they utilize. The number of hydrogen bond donors appeared to have positive correlation with the number of virtual hits for all transforms. The strongest correlations were found for transform 7021 (Horner–Wadsworth–Emmons olefination). Hydrogen bonds are strong contributors to the binding energy. Thus, hydrogen bond-forming capacity of the pocket can be expected to have a positive effect on the number of virtual hits. However, as discussed before, prefiltering of the building blocks for “drug-like” properties, which excludes hydrogen bond donor-rich compounds to avoid cell permeability and bioavailability issues, limits the number of HBD-rich compounds, making the observed dependences less pronounced. The hydrogen bond forming capacity of a transform can be impacted by reaction selectivity. For example, both transform 7013 and 7014 generate benzimidazoles from aromatic o-diamines and aldehydes. However, 7014 uses boric acid to produce a reactive intermediate while 7013 uses molecular iodine under basic conditions. Consequently, the sets of restrictions for the starting blocks are different. As a result, 7013 generated almost twice as many compounds as 7014, but has a significantly lower overall virtual hit rate (Table 1). Nevertheless, the correlations with pocket properties are similar for these two transforms. All observed trends can assist in generation of optimally targeted virtual libraries and thus, reduce time and effort required for lead identification.

## Conclusions

The results show that chemistries used for preparation of virtual libraries have substantial impact on the virtual hit rates during virtual screening. The data suggest that with the ever-expanding number of synthetically accessible compounds and limited computational resources, efforts in enumeration of virtual libraries will benefit from focusing on cross-coupling reactions such as Sonogashira, Suzuki–Miyaura, Hiyama and Liebeskind–Srogl couplings as they produce the highest numbers of virtual hits for different types of protein pockets. Transforms 1439 (pyrazoles synthesis from beta carbonyl carboxylic acid derivatives), 2238 (synthesis of fused aryl(2,3-H/R) pyridines by Pictet-Spengler reaction, 2269 (Kabbe synthesis of 4-chromanones, 6032 (Mitsunobu carbon–carbon bond formation), and 7018 (sulfonamide acylation) were among the least effective ones in generation of virtual hits for all tested pockets, and thus, can be given a lower priority in generation of custom libraries.

For larger pockets, transforms 2201, 6003, 6004, 6005, 7013 and 7014 appeared to be most effective.

The DLID (drug-like density) descriptor had a positive correlation with the number of virtual hits. The correlation was statistically significant for the entire diversity set and for 10 transforms out of 53. The lack of significant correlation for most of transforms suggests that low druggability score, in its traditional definition [37] should not discourage one from attempting virtual screens for a particular pocket as exceptions to the rule are not uncommon.

## Methods

### Databases

The SAVI diversity set of 2,955,416 compounds was generated from the entire SAVI-2020 database (which contains 1,748,464,003 compounds), using mini-batch k-means clustering performed with RDKit [42] and scikit-learn [43]. The Tanimoto coefficient for any two compounds in the set was < 0.6. The entire SAVI database and diversity sets are available for downloading from the SAVI download page [27].

### Database docking

Docking screens were conducted using the ICM-Pro software (Molsoft L.L.C., San Diego, CA) by running 590 parallel processes (5000 compounds per job) on 590 nodes of the National Institutes of Health (NIH) Biowulf cluster supercomputer [44]. Each node contained 2 CPUs. The PocketFinder software (Molsoft) was used for the identification of the pockets. Screens were run in large-scale parallel way as so-called "swarm" jobs. The cutoff score to identify a virtual hit was set to -32 for the pockets that produced redocking scores lower than -32 for the “native” ligands of the complexes from Protein Data Bank or had no available structures of the complexes (Table 1). For the structures that produced re-docking score higher than −32 (PDBs: 3odu, 5i96, 5fhz, 4xe0, 5vv0, 5kj2, and 4ixd, Table 1) their redocking scores have been used as cutoffs. Virtual hits were extracted as Excel files. Every compound in the SAVI database has an identifier (SAVI ID) with its last four digits indicating the transform number. These numbers were used for counting virtual hits produced by every transform. Correlation coefficients, Student's t-distribution and p-values were determined using the Data Analysis function of Excel (Microsoft).

## Supplementary Information

Below is the link to the electronic supplementary material.

Supplementary file1 (XLSX 51 KB)

## Data Availability

All data generated or analyzed during this study are included in this published article and its supplementary information. The databases used in the study are freely available from NCI webpage [27].
